# Impact of glycemic control status on patients with ST-segment elevation myocardial infarction undergoing percutaneous coronary intervention

**DOI:** 10.1186/s12872-020-01339-x

**Published:** 2020-01-30

**Authors:** Yan Li, Xiaowen Li, Yinhua Zhang, Leimin Zhang, Qingqing Wu, Zhaorun Bai, Jin Si, Xuebing Zuo, Ning Shi, Jing Li, Xi Chu

**Affiliations:** 1 Departent of Cardiology, Xuanwu hospital, Capital Medical University, Beijing, China; 2 Emergency Department, Aerospace Center Hospital, Beijing, China; 3 Department of internal medicine, Qinghe Substation Hospital of Beijing Municipal Administration of Prisons, Beijing, China; 4 Department of Cardiology, Beijing Luhe Hospital, Capital Medical University, Beijing, China; 5 Health Management Center, Xuanwu hospital, Capital Medical University, Beijing, China

**Keywords:** ST-segment elevation myocardial infarction, Hyperglycemia, Diabetes, Glycated hemoglobin, Percutaneous coronary intervention

## Abstract

**Background:**

The combined effects of diabetes mellitus (DM), admission plasma glucose (APG), and glycated hemoglobin (HbA1c) levels on predicting long-term clinical outcomes in patients with ST-segment elevation myocardial infarction (STEMI) undergoing primary percutaneous coronary intervention (pPCI) are unknown. Therefore, we evaluated their combined effects on long-term clinical outcomes in STEMI patients treated with pPCI.

**Methods:**

In total, 350 consecutive patients with STEMI undergoing pPCI were enrolled. Patients were divided into 3 groups according to DM history and APG and HbA1c levels. The cumulative rates of 24-month all-cause deaths and major adverse cardiac and cerebrovascular events (MACCEs) were calculated.

**Results:**

Both the incidence of all-cause deaths and cumulative rates of MACCEs were significantly the lowest in patients without a DM history and admission HbA1c level < 6.5%. DM patients with poor glycemic control or stress hyperglycemia on admission experienced the highest rates of all-cause deaths, MACCEs, and cardiac deaths. Admission HbA1c levels, Triglyceride (TG) levels, hemoglobin levels, DM history, and admission Killip class > 1 correlated with 24-month all-cause death; HbA1c levels on admission, DM history, APG levels, history of stroke, history of coronary heart disease, and TG levels on admission were significantly associated with MACCEs through the 24-month follow-up. The predictive effects of combining DM and APG and HbA1c levels were such that for STEMI patients undergoing pPCI, DM patients with poor glycemic control or with stress hyperglycemia on admission had worse prognosis than other patients.

**Conclusion:**

Strict control of glycemic status may improve the survival of patients who have both DM and coronary heart diseases.

## Background

Ischemic heart disease threatens global health and leads to increasing mortality worldwide [1]. In 1977, the first percutaneous coronary intervention (PCI) was performed. Currently, PCI has become one of the most frequently performed therapeutic interventions in acute myocardial infarction (AMI) cases, resulting in a steady decline in periprocedural adverse events [2]. Diabetic patients account for more than a quarter of all patients undergoing PCI [3, 4]. Diabetes mellitus (DM) has long been recognized as an independent risk factor for cardiovascular disease (CVD) and is also an independent predictor of adverse clinical outcomes after PCI [5, 6]. Hyperglycemia on admission has been associated with major adverse cardiovascular events (MACEs) and increased mortality in patients admitted with AMI. It has also been associated with higher in-hospital and long-term mortality in patients with ST-segment elevation myocardial infarction (STEMI) undergoing primary PCI (pPCI) [7–10]. Since 2010, glycated hemoglobin (HbA1c) has been recommended by the World Health Organization and American Diabetes Association as a point-of-care test for the diagnosis of DM (≥6.5%) [11]. HbA1c is also a marker of glycemic control status for the previous 8 to 12 weeks; an elevated HbA1c level is associated with an increased risk of cardiovascular diseases in patients with DM [12]. DM, admission plasma glucose (APG), and HbA1c levels are associated with clinical outcomes in patients with CVD. Nevertheless, the combined effects of DM, APG, and HbA1c on clinical outcomes in Chinese patients with STEMI undergoing pPCI remain unknown, and if the combined effects can be clearly elucidated, the management of patients with coronary heart disease (CHD) can be improved, and blood sugar control can be more accurately emphasized. Therefore, we evaluated the combined effects of DM and APG and HbA1c levels on long-term clinical outcomes in STEMI patients treated with pPCI.

## Methods

This retrospective study investigated the combined effects of DM and APG and HbA1c levels on long-term clinical outcomes in STEMI patients treated with pPCI. Patients with STEMI undergoing pPCI in Xuanwu Hospital Capital Medical University from April 2009 to December 2015 were enrolled in our study. Patients were eligible if they had both of the following: 1) an elevated cardiac troponin I level (≥2.0 ng/mL) or troponin T level (≥0.1 ng/mL) or creatine kinase-MB (≥19 U/L, exceeding twice the upper limit of normal) and 2) new ST-segment elevation of greater than 2 mm in at least 2 precordial leads or greater than 1 mm in at 2 least limb leads. Patients were excluded if 1) there were no data on APG and admission HbA1c levels; 2) they had a history of coronary artery bypass grafting; or 3) they were lost to follow-up. The follow-up information of patients was collected by medical records or telephone contact with the patient at 6th and 12th months, and then annually. The shortest follow-up time was 24 months. The cumulative rates of 24-month all-cause death and major adverse cardiac and cerebrovascular events (MACCEs) were assessed as adverse clinical outcomes. This study was approved by the Ethics Committee of the Xuanwu Hospital Capital Medical University. All participants signed informed consent forms.

DM was defined as having a previous history of type 2 DM based on medical institution standard diagnostic criteria, use of diet, oral glucose-lowering medication, and/or insulin or an HbA1c level ≥ 6.5% [11]. Previous studies found that the prognostic cut-off between hyperglycemia and adverse clinical outcomes in individuals with DM [7, 13, 14] was 180 mg/dL (10 mmol/L) for those with DM, as shown in a meta-analysis by Capes et al. [8]. The HbA1c levels of non-pregnant adults with DM should be < 7.0% according to the recommendation of Standards of Medical Care in Diabetes-2018 [15]. When patients have hyperglycemia in hospital, they were accepted insulin therapy to decrease their plasma glucose.

According to these criteria, 350 patients were divided into 3 groups: Group 1 (*n* = 174) with no DM and admission HbA1c level < 6.5%; Group 2 (*n* = 64) with DM, good glycemic control, and no stress hyperglycemia on admission, defined as follows: a history of DM or admission HbA1c level ≥ 6.5% or APG < 10 mmol/L and admission HbA1c < 7%; and Group 3 (*n* = 112) with DM but not good glycemic control or stress hyperglycemia on admission, defined as follows: a history of DM or admission HbA1c level ≥ 6.5%, APG level ≥ 10 mmol/L, or admission HbA1c ≥7%.

The primary outcomes were 24-month cumulative all-cause deaths and MACCEs. MACCEs include cardiac deaths, stent thrombosis, repeat revascularization, myocardial infarction (MI), and stroke.

Continuous variables are described as mean ± standard deviation or median (interquartile range), and differences among the groups were assessed by the independent t-test or the Wilcoxon rank-sum test. Categorical variables were described as number (n) with percentage (%), and differences were analyzed by the chi-square test or Fisher exact test. Predictors of 24-month cumulative all-cause deaths and MACCEs were identified using a multivariable Cox regression analysis. We included covariates that were statistically significant in univariate analysis or those that were clinically relevant in the multivariate analysis: a history of DM, history of CHD, hypertension, history of stroke, multivessel disease, admission Killip class, admission laboratory results (glucose, HbA1c, triglyceride [TG], high-density lipoprotein, low-density lipoprotein, apolipoprotein AI, apolipoprotein B [Apo B] and hemoglobin levels), and long-term medication before admission (regular medication usage over a 6-month period; e.g., aspirin, statin, beta-blocker, angiotensin-converting enzyme inhibitor [ACEI]). Kaplan-Meier methods were used to estimate the rates of 24-month cumulative all-cause deaths and MACCEs and to plot the time-to-cumulative occurrence of all-cause deaths and MACCEs in the 3 groups. The significance of differences among the groups was determined using the log-rank test. SPSS version 22.0 (IBM Corp., Chicago, IL) was used for statistical analysis, and a *P* value < 0.05 was defined as the threshold of statistical significance.

## Results

Among 350 patients, 176 (50.3%) met the criteria for DM (Groups 2 and 3). The baseline characteristics and angiographic findings are listed in Table 1. Patients with DM had higher rates of hypertension and were taking beta-blockers long-term before admission compared to patients without DM (*P* < 0.05). Patients in Group 2 had higher rates of long-term aspirin use before admission than did patients in Group 1 (*P* < 0.05). Compared with Group 1 patients, Group 3 patients were older, had more men, a higher number of patients with multivessel disease, higher long-term ACEI use before admission, lower hemoglobin level and higher admission Killip class (*P* < 0.05). Group 3 patients had the highest rates of insulin therapy in hospital and the highest values of admission TG and Apo B levels (*P* < 0.05).

**Table 1:** Baseline characteristics

Characteristic	Group 1 (174)	Group 2 (64)	Group 3 (112)
Age (years)	59.34 ± 11.71	61.61 ± 12.85	64.29 ± 10.84 ^#^
Male (%)	149 (85.6%)	52 (81.3%)	80 (71.4%)^#^
Prior CHD (%)	30 (17.2%)	15 (23.4%)	30 (26.8%)
Hypertension (%)	80 (46%)	43 (67.2%)^#^	66 (58.9%)^#^
Prior Stroke (%)	16 (9.2%)	9 (14.1%)	13 (11.6%)
Prior PCI (%)	12 (6.9%)	8 (12.5%)	15 (13.3%)
Multivessel disease	90 (51.8%)	42 (65.6%)	88 (78.6%)^#^
Killip class 3–4 (%)	8 (4.6%)	3 (4.6%)	12 (10.7%) ^#^
Laboratory results			
Glucose (mmol/L)	5.69 (5.05–6.60)	6.65 ^#^ (6.15–7.97)	11.11 ^# *^ (7.85–13.76)
HbA1c (%)	5.60 (5.30–5.90)	6.60 ^#^ (6.10–6.90)	7.80 ^# *^ (7.30–9.58)
Hemoglobin(g/L)	148.63 ± 15.05	144.94 ± 16.58	143.36 ± 17.26^#^
TG (mmol/L)	1.46 (1.07–2.04)	1.51 (1.04–2.10)	1.70 ^# *^ (1.22–2.30)
HDL-C (mmol/L)	1.24 (0.99–1.47)	1.23 (0.99–1.46)	1.20 (0.99–1.43)
LDL-C (mmol/L)	2.72 (2.20–3.33)	2.73 (2.22–3.35)	2.77 (2.26–3.35)
Apo AI (g/L)	1.14 (0.96–1.33)	1.17 (0.99–1.35)	1.17 (1.01–1.24)
Apo B (g/L)	0.84 (0.68–0.97)	0.85 (0.69–0.97)	0.88 ^# *^ (0.79–1.02)
Creatinine(umol/L)	68.00 (61.00–78.25)	68.50 (61.00–80.75)	67.50 (57.00–79.75)
Long-term medication before admission			
Aspirin	21 (12.5%)	17 (27.4%)^#^	20 (17.9%)
Clopidogrel	12 (7.1%)	7(11.3%)	11 (9.8%)
Statin	11 (6.5%)	9 (14.5%)	16 (14.2%)
Beta-blocker	16 (9.5%)	12 (19.4%)^#^	21 (18.8%)^#^
ACEI	11 (6.5%)	5 (8.1%)	17 (15.1%) ^#^
Medication in hospital			
Insulin	19(10.9%)	10(15.6%)	63(56.3%) ^# *^
Beta-blocker	155(89.1%)	58(90.6%)	101(90.2%)
ACEI	155(89.1%)	62(96.9%)	106(94.6%)

*ACEI* angiotensin converting enzyme inhibitor, *Apo AI* apolipoprotein AI, *Apo B* apolipoprotein B, *CHD* coronary heart disease, *HbA1c* glycated hemoglobin, *HDL* high-density lipoprotein, *LDL* Low-density lipoprotein, *PCI* percutaneous coronary intervention, *TG* triglyceride

^#^*P* < 0.05, vs Group 1

^*^P < 0.05, vs Group 2

**Fig. 1:**
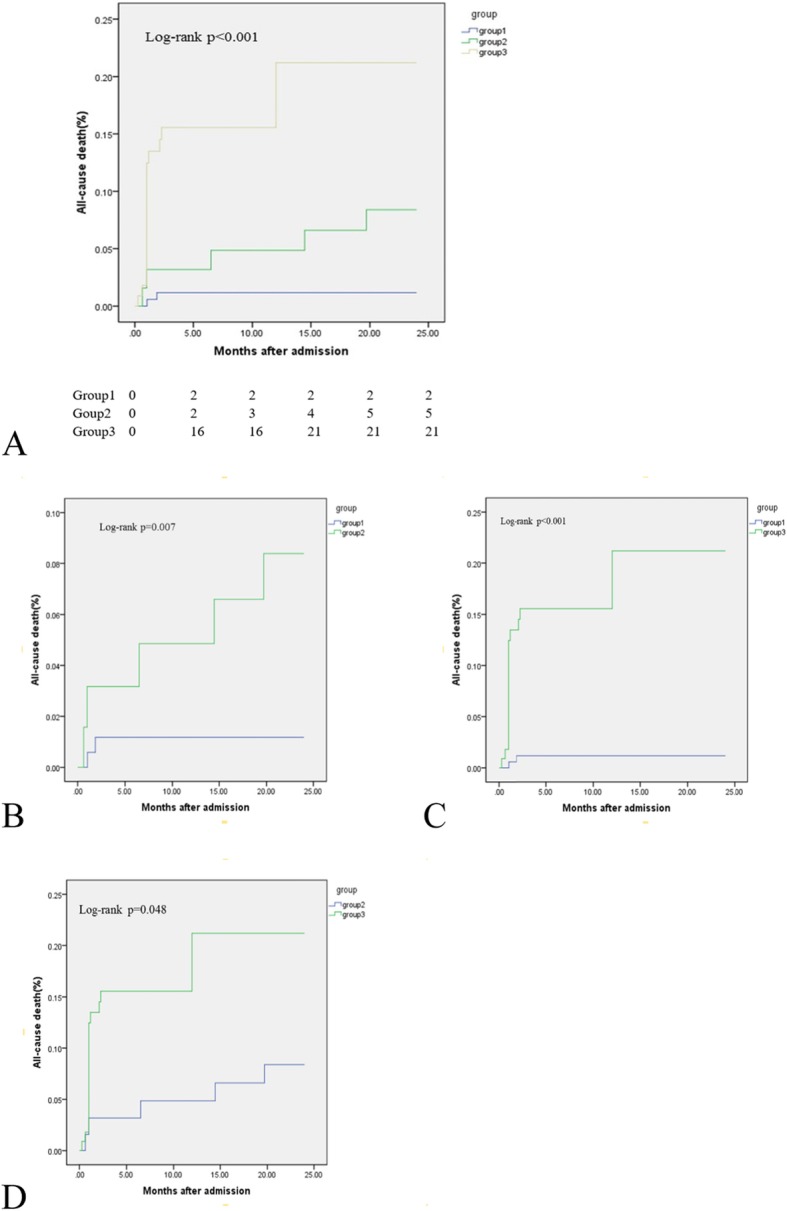
Comparison of all-cause death rates in the overall population. **a** Comparison among the 3 groups; (**b**) comparison of Groups 1 and 2; (**c**) comparison of Groups 1 and 3; and (**d**) comparison of Groups 2 and 3

**Fig. 2:**
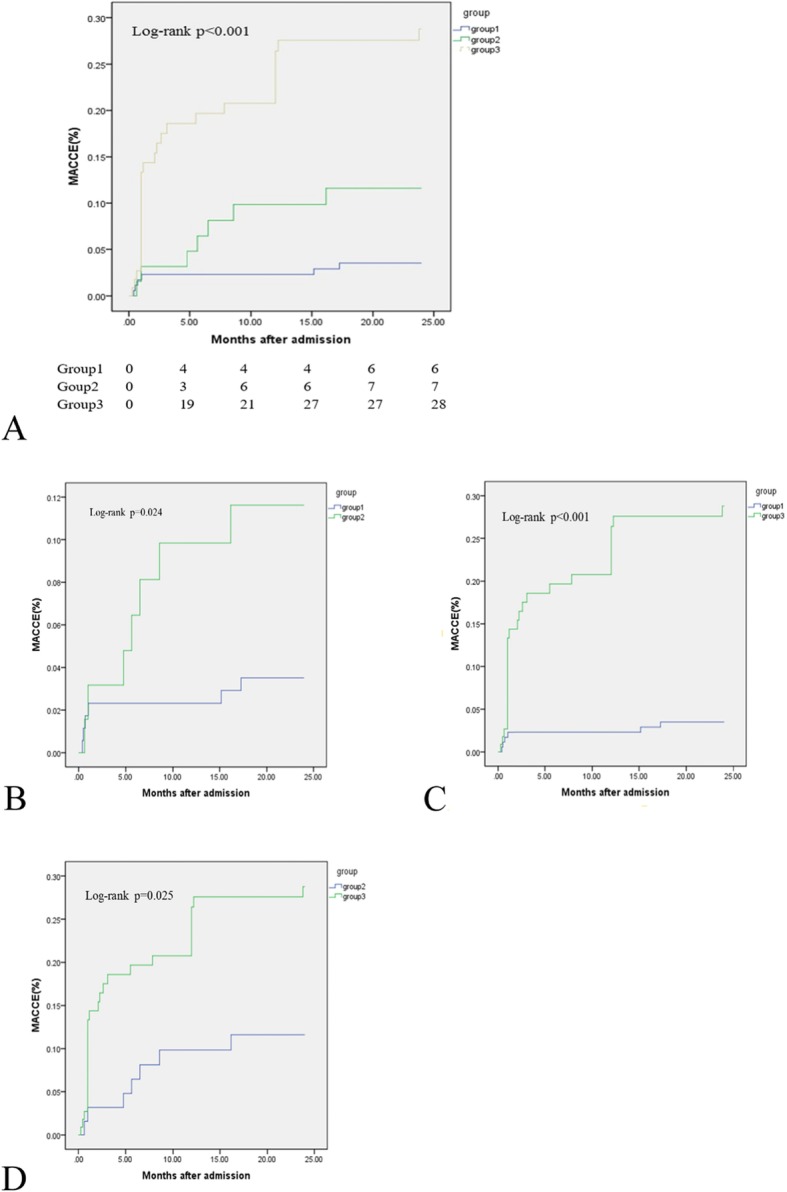
Comparison of MACCE rates in the overall population. **a** Comparison among the 3 groups, (**b**) comparison of Groups 1 and 2; (**c**) comparison of Groups 1 and 3; and (**d**) comparison of Groups 2 and 3. MACCEs, major adverse cardiac and cerebrovascular events including cardiac death, stent thrombosis, repeat revascularization, MI, and stroke; MI, myocardial infarction

**Fig. 3:**
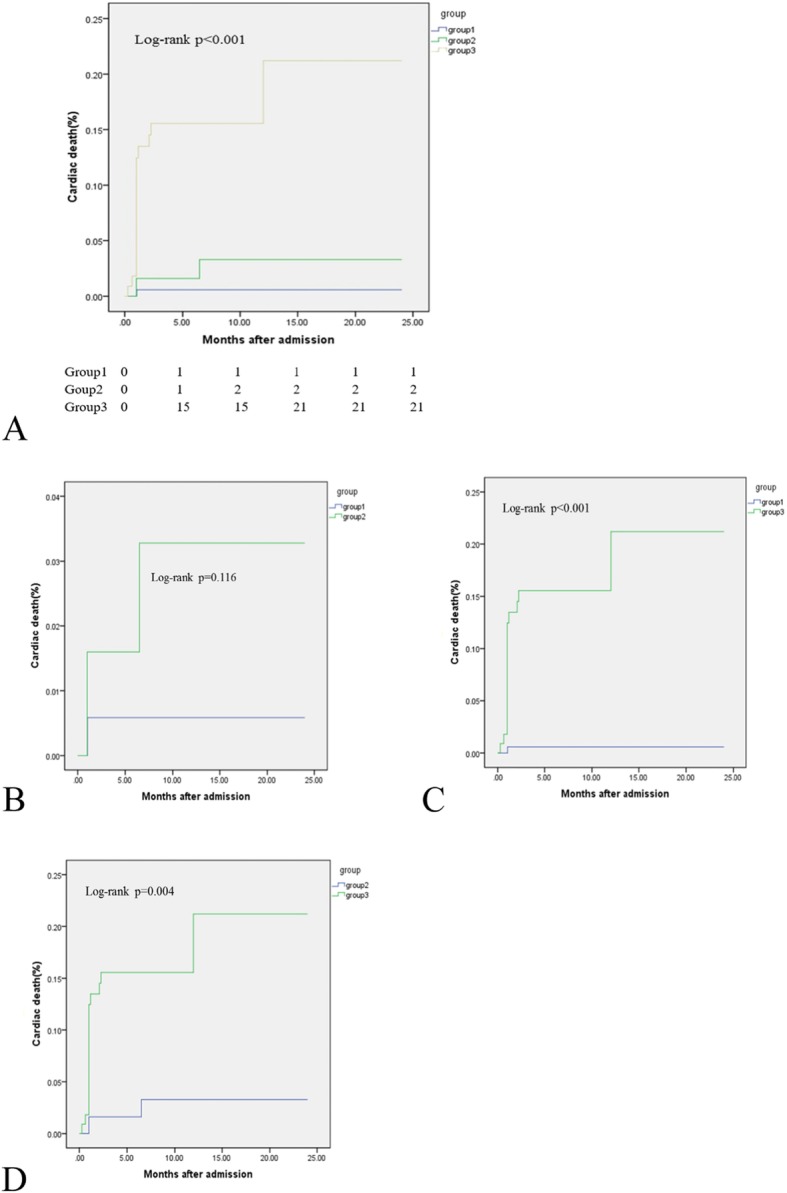
Comparison of cardiac death rates in the overall population. **a** Comparison among 3 groups, (**b**) comparison of Groups 1 and 2; (**c**) comparison of Groups 1 and 3; and (**d**) comparison of Groups 2 and 3

At the 24-month follow-up, 28 patients died, and 41 patients experienced MACCEs. The Kaplan-Meier survival curves showed that both the incidence of all-cause death and the cumulative rates of MACCEs were the lowest in Group 1, and Group 3 patients had the highest rates of all-cause death, MACCEs, and cardiac death (*P* < 0.05) **(**Table 2**)** (Figs. 1, 2 and 3).

**Table 2:** Clinical outcomes at 24-month follow-up

Adverse Events	Group 1 (174)	Group 2 (64)	Group 3 (112)	*P* value			
				overall	1 vs 2	1 vs 3	2 vs 3
All-cause death	2 (1.1%)	5 (7.8%)	21 (18.8%)	< 0.001	0.007	< 0.001	0.048
MACCEs	6 (3.4%)	7 (10.9%)	28 (25%)	< 0.001	0.024	< 0.001	0.025
Cardiac death	1 (0.6%)	2 (3.1%)	21 (18.8%)	< 0.001	0.116	< 0.001	0.004
Stent thrombosis	2 (1.1%)	0 (0)	1 (0.9%)	0.7	0.390	0.874	0.418
Repeat revascularization	2 (1.1%)	4 (6.3%)	4 (3.6%)	0.085	0.024	0.111	0.532
MI	2 (1.1%)	0 (0)	3 (2.7%)	0.264	0.403	0.268	0.170
Stroke	0 (0)	1 (1.6%)	1 (0.9%)	0.296	0.161	0.161	0.776

Values expressed are n (%). *MACCEs* major adverse cardiac and cerebrovascular event, includes cardiac death, stent thrombosis, repeat revascularization, MI and stroke, *MI* myocardial infarction

Admission HbA1c levels, TG levels, hemoglobin levels, history of DM, and admission Killip class > 1 were significantly associated with all-cause death through the 24-month follow-up (*P* < 0.05). The incidence of 24-month MACCEs significantly correlated with admission HbA1c levels, APG levels, TG levels, history of DM, stroke, and CHD (*P* < 0.05) (Tables 3 and 4**)**.

**Table 3:** The relationship between 24-month all-cause death outcomes and risk factors

Variable	HR	95% CI	*P* value
Admission HbA1c	1.283	[1.056, 1.558]	0.012
Prior DM	4.402	[1.438, 13.469]	0.009
Admission Killip class 2–4	2.906	[1.182, 7.143]	0.020
Admission Hemoglobin	0.955	[0.927, 0.984]	0.003
Admission TG	1.048	[1.026, 1.070]	< 0.001

*CI* confidence interval, *DM* diabetes mellitus, *HbA1c* glycated hemoglobin, *HR* hazard ratio, *TG* triglyceride

**Table 4:** The relationship between 24-month MACCEs outcomes and risk factors

Variable	HR	95% CI	*P* value
Admission HbA1c	1.353	[1.142, 1.603]	< 0.001
Prior DM	3.372	[1.483, 7.664]	0.004
Admission glucose (high tertile (> 9.295 mmol/L))	2.595	[1.267, 5.312]	0.009
Prior stroke	4.136	[1.803, 9.490]	0.001
Prior CHD	2.839	[1.195, 6.742]	0.018
Admission TG	1.050	[1.030, 1.071]	< 0.001

*CHD* coronary heart disease, *CI* confidence interval, *DM* diabetes mellitus, *HbA1c* glycated hemoglobin, *HR* hazard ratio, *MACCEs* major adverse cardiac and cerebrovascular events, including cardiac death, stent thrombosis, repeat revascularization, MI and stroke, *TG* triglyceride

## Discussion

We demonstrated that patients without DM had a better prognosis after PCI than patients with DM undergoing PCI in terms of 24-month all-cause death and MACCEs. De Luca et al. [16] also found that patients with DM were more likely to have poor prognostic outcomes and a higher incidence of adverse events. A meta-analysis declared that in-hospital, short-, and long-term mortality was occurred apparently higher in diabetic patients after PCI, respectively, than in non-diabetic counterparts [17]. Therefore, in patients undergoing PCI, DM is an independent risk subset associated with worse clinical outcomes. Patients with DM were more likely to have higher rates of left main stenosis, chronic total occlusions, diffuse and multivessel disease, smaller vessel sizes, and longer lesion lengths [18, 19]. All these factors may affect subsequent revascularization. Furthermore, greater plaque burden, higher propensity for plaque rupture [20], enhanced prothrombotic status, exuberant neointimal hyperplasia [21], more aggressive pattern of atherosclerosis, and endothelial dysfunction are seen in the inflammatory environments in patients with DM [22]. All these data suggest that patients with DM experience a higher number of adverse events.

Many studies demonstrated that APG was an indicator of the risk of short- and long-term MACCEs in patients undergoing PCI [8, 23–25]. We also found that higher APG levels were associated with higher rates of 24-month MACCEs by multivariate Cox regression analysis, regardless of the diagnosis of DM. Hyperglycemia on admission was considered as an acute stress response. Some investigations found that the impact of acute hyperglycemia seems to be more pronounced in patients without DM than in those with DM, suggesting that the magnitude of the acute glycemic rise from chronic levels, rather than the absolute admission glycemic level, can be detrimental [26–28]. When DM patients have hyperglycemia, the hyperglycemia can be derived from acute stress response or bad glycemic control before admission. Because of oxidative stress and amplified inflammatory immune reactions, stress hyperglycemia after STEMI could lead to endothelial and microvascular dysfunction. Plasma glucose levels are related to circulating inflammatory cytokine levels positively. Intensified oxidative stress and damaged endothelial function may be caused by higher levels of inflammatory cytokine concentrations, whereas reductions in circulating levels of inflammatory cytokines can improve endothelial function [29, 30]. Furthermore, hyperglycemia would provoke prothrombotic state and then decrease plasma fibrinolytic activity and activated tissue plasminogen [31]. According to these mechanisms, impaired myocytes [31, 32], impaired left ventricular function, and exacerbated cardiac damage may come from stress hyperglycemia after STEMI [33]. Taken together, these data suggest that admission hyperglycemia could be a negative prognostic factor among patients with STEMI. When the admission hyperglycemia in DM patients derive from bad glycemic control, our investigation also found STEMI patients with bad glycemic control before admission according to HbA1c have higher 24-month all-cause deaths and MACCEs rates. A meta-analysis that enrolled 33,040 participants reported that a 0.9% decline in the HbA1c level was associated with a 17% decrease in MACEs during acute coronary syndrome in patients with DM [34]. When we inputted admission HbA1c levels into the Cox regression proportional hazard multivariate analysis for all-cause deaths and MACCEs outcomes at 24 months, we found that higher admission HbA1c levels were significantly associated with higher rates of all-cause deaths and MACCEs. There are several possible mechanisms to explain the associations between higher HbA1c levels and poor clinical outcomes. First, Increased HbA1c is a measurement of previous poor glycemic control, and there is evidence that chronic hyperglycemia can induce vascular endothelial cell damage, with resulting vasomotor dysfunction, excessive extracellular matrix formation, and increased cellular proliferation [35], all of which can lead to adverse clinical outcomes after PCI. Second, Saleem et al.’s study found that the HbA1c level was an independent factor influencing the severity of CAD, as demonstrated by coronary angiography [36]. Third, an increase in HbA1c levels was clearly associated with adverse baseline characteristics such as a higher cardiovascular risk profile, and this study may partly explain the increase in long-term deaths [37].

When we place DM history, APG levels, and admission HbA1c levels together as a combined marker, we found that STEMI patients without DM after pPCI had the best prognosis, and DM patients with bad glycemic control or with stress hyperglycemia on admission had the worst prognosis in terms of 24-month all-cause deaths and MACCEs. We propose considering this combined marker as a long-term prognostic marker that can estimate adverse clinical outcome rates after pPCI for STEMI, and our study also helps direct blood glucose management of patients with STEMI undergoing PCI. On the basis of our study findings, we recommend that patients with STEMI should control their blood glucose strictly whether or not they have been diagnosed with DM.

The present study has some limitations. First, this was a single-center observational study that included a relatively small sample size, which may lead to data bias; thus, a larger sample and more studies are needed to verify our results. Second, there were no considerations of the associations of impaired glucose tolerance or postprandial hyperglycemia with an increased risk for coronary artery disease because we did not perform oral glucose tolerance test (OGTT). We suggest that patients undergo OGTT if their admission blood glucose is beyond the range of normal values. Thirdly, our investigation was retrospective, and we only include STEMI patients and all of DM patients were type 2 DM. NSTEMI patients undergoing PCI and type 1 DM patients should also be investigated. Fourthly, our database doesn’t have data on patients’ body mass index (BMI), TIMI flow after pPCI, left ventricular ejection fraction, length of DM and therapy after discharge. These data are important to patients’ prognosis.

## Conclusions

DM and higher APG and admission HbA1c levels led to higher rates of 24-month MACCEs in Chinese patients with STEMI undergoing pPCI. The combined effects of DM, APG levels, and HbA1c levels demonstrated that patients without DM had the best prognosis. Patients with both DM and CHD should control their glycemic levels strictly; this may improve their survival over that of patients with poor glycemic control or stress hyperglycemia on admission.

## Data Availability

The data used to support the findings of this study are available from the corresponding author upon reasonable request.

## Abbreviations

ACEI: Angiotensin-converting enzyme inhibitor
AMI: Acute myocardial infarction
APG: Admission plasma glucose
Apo B: Apolipoprotein B
CHD: Coronary heart disease
CVD: Cardiovascular disease
DM: Diabetes mellitus
HbA1c: Glycated hemoglobin
MACCEs: Major adverse cardiac and cerebrovascular events
MACEs: Major adverse cardiovascular events
MI: Myocardial infarction
OGTT: Oral glucose tolerance test
PCI: Percutaneous coronary intervention
STEMI: ST-segment elevation myocardial infarction
TG: Triglyceride
